# ESCRT-III mediated cell division in *Sulfolobus acidocaldarius* – a reconstitution perspective

**DOI:** 10.3389/fmicb.2014.00257

**Published:** 2014-06-04

**Authors:** Tobias Härtel, Petra Schwille

**Affiliations:** Department of Cellular and Molecular Biophysics, Max Planck Institute of BiochemistryMartinsried, Germany

**Keywords:** *Sulfolobus acidocaldarius*, cell division, ESCRT-III, Vps4, CdvA, GUV

## Abstract

In the framework of synthetic biology, it has become an intriguing question what would be the minimal representation of cell division machinery. Thus, it seems appropriate to compare how cell division is realized in different microorganisms. In particular, the cell division system of Crenarchaeota lacks certain proteins found in most bacteria and Euryarchaeota, such as FtsZ, MreB or the Min system. The *Sulfolobaceae* family encodes functional homologs of the eukaryotic proteins vacuolar protein sorting 4 (Vps4) and endosomal sorting complex required for transport-III (ESCRT-III). ESCRT-III is essential for several eukaryotic pathways, e.g., budding of intraluminal vesicles, or cytokinesis, whereas Vps4 dissociates the ESCRT-III complex from the membrane. Cell Division A (CdvA) is required for the recruitment of crenarchaeal ESCRT-III proteins to the membrane at mid-cell. The proteins polymerize and form a smaller structure during constriction. Thus, ESCRT-III mediated cell division in *Sulfolobus acidocaldarius* shows functional analogies to the Z ring observed in prokaryotes like *Escherichia coli*, which has recently begun to be reconstituted *in vitro*. In this short perspective, we discuss the possibility of building such an *in vitro* cell division system on basis of archaeal ESCRT-III.

## ARCHAEAL CELL DIVISION

Although the basic requirements for any cell division are evident: (a) Identification of a division site, (b) targeting and assembly of elements required for membrane and cell wall constriction, and (c) coordination of constriction with genome segregation, we still lack a fundamental quantitative understanding of these processes in nearly all organisms. This can be exemplified by the open questions of how large the constricting forces actually have to be, and what brings them about. Obviously, at least the last step of membrane constriction involves membrane fusion, constituting an obvious link between division and membrane fusion machineries in general and potentially across species. Notably, recent works revealed the important roles of the archaeal homologs to the eukaryotic vacuolar protein sorting 4 (Vps4, Saci_1372, also called CdvC) and endosomal sorting complex required for transport-III (ESCRT-III, Saci_1373, also called CdvB) proteins together with the protein CdvA (Saci_1374) for cell division in *Sulfolobus acidocaldarius* (Obita et al., 2007; Lindas et al., 2008; Samson et al., 2008, 2011; Wollert et al., 2009a, b; Wollert and Hurley, 2010) and for secretion of vesicles (Ellen et al., 2009). After the segregation of the nucleoids, a ring formed of CdvA is supposed to precede the appearance of the ESCRT-III rings, which are later disassembled by the Vps4 (Samson et al., 2011). This raises the important question whether these protein machineries may be potential candidates as constriction force generators, and for reconstituting coordinated membrane constriction with a minimal set of components, irrespective of scaling issues. In the following, we will briefly discuss the relevant modules of this machinery and their known functions.

## CdvA, A CYTOSKELETAL LINK BETWEEN DNA, MEMBRANE, AND ESCRT-III

The membrane-binding cytoskeletal protein CdvA was first investigated in *Metallosphaera sedula* and exhibits functional homologs that are only expressed in Crenarchaeota (except Thermoproteales) and Thaumarchaeota (Moriscot et al., 2011; Samson et al., 2011). Phylogenetic analysis in NCBI databases led to a tree that is notably homogeneous within Archaea, indicating that the distribution of CdvA is no horizontal gene transfer (HGT) event (Moriscot et al., 2011), and is evolutionary lost in Thermoproteales and possibly also in Euryarchaeota (Moriscot et al., 2011).

**FIGURE 1:**
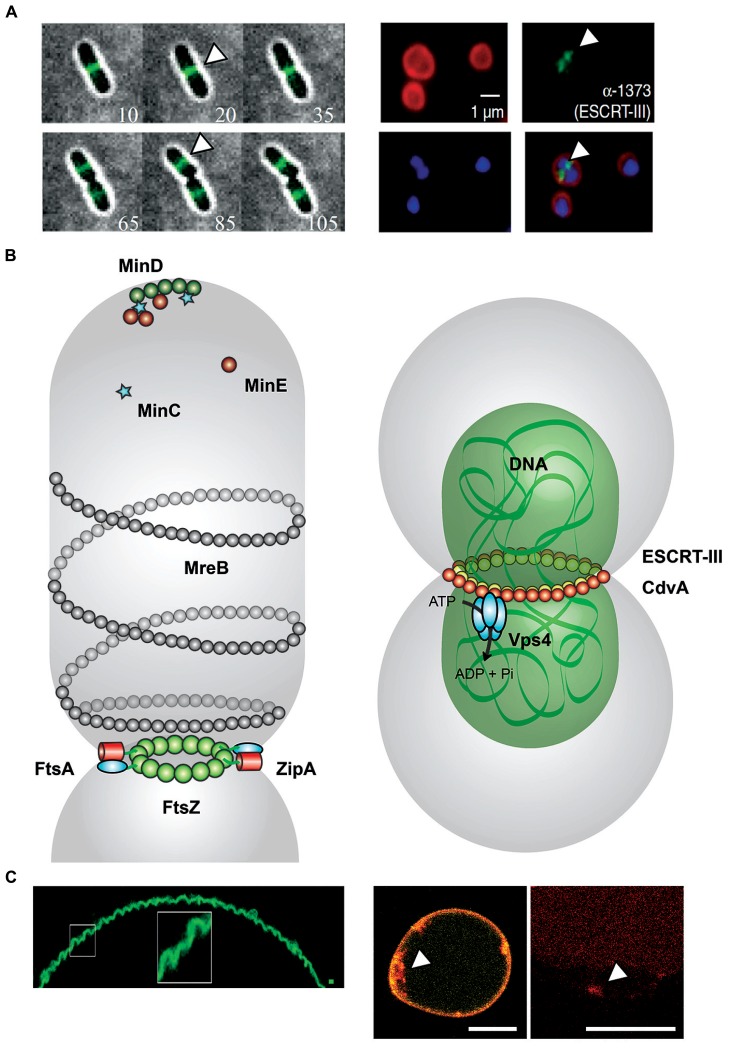
**Comparison between FtsZ- and ESCRT-III mediated cell division. (A)** Left: Montages from time-lapse movies of *Escherichia coli* cells expressing FtsZ–GFP during cell division. At each time point, the fluorescence image (green) was overlaid with the corresponding bright-field image (gray) and the time (in minutes) displayed in the bottom corner. FtsZ (white arrowheads) forms a clear band at mid-cell early and remains there throughout the division process (Buss et al., 2013). Right: Localization of ESCRT-III in *Sulfolobus acidocaldarius*. Representative images show the FM4-64X staining for membrane (red), DAPI staining for DNA (blue), antibody labeling of ESCRT-III, and merged images. ESCRT-III localization is visualized by white arrowheads. Scale bar, 1 μm (Samson et al., 2008). **(B)** Left: FtsZ-mediated cell division in *Escherichia coli*. During the initial stage of proto-ring assembly, FtsZ (green) is located to mid-cell by the oscillating Min waves [MinD (dark green), MinE (dark red), and MinC (light blue stars)]. FtsZ attaches to the membrane-associated FtsA (cyan). ZipA (red) binds competitive with FtsA to FtsZ. ZipA–FtsZ binding also increase membrane fluidity (Rico et al., 2013). MreB (gray) structures colocalize with Z ring at mid-cell (Fenton and Gerdes, 2013). Right: ESCRT-III-mediated cell division in *Sulfolobus acidocaldarius*. CdvA (Saci_1374, yellow) and DNA (green) build up double-helical structures horizontally to the cytokinesis region in *Sulfolobus acidocaldarius* (gray). ESCRT-III (Saci_1373, red) and paralogs interact with CdvA via its C-terminal winged Helix-like domain and also bind to MIT domain of the Vps4 (Saci_1372, cyan). The AAA-type ATPase Vps4 regenerates the ESCRT-III complex after cytokinesis (Moriscot et al., 2011; Samson et al., 2011). **(C)** Left: Membrane curvature induced in GUVs by assembly of FtsZ filaments. Under low membrane tension conditions, MTS-FtsZ-YFP showed spontaneous deformation of the GUV membranes (Arumugam et al., 2012). Right: Membrane curvature events in GUVs by the ESCRT-III machinery. By droplet emulsion transfer method CdvA, ESCRT-III (red Alexa647 labeled) and Vps4 were inserted into DOPC GUVs (yellow). The location of ESCRT-III (white arrowhead) to curved membrane suggests a function in membrane deformation event. Scale bar, 10 μm.

In *Sulfolobus acidocaldarius*, CdvA (Saci_1374) serves as a recruitment platform for ESCRT-III (Lindas et al., 2008; Moriscot et al., 2011; Samson et al., 2011). The CdvA/ESCRT-III interface revealed a novel interaction site between the C-terminal winged Helix-like domain of crenarchaeal ESCRT-III and CdvA, which also impacts on the MIT domain of Vps4 in a similar way as in eukaryotes (Obita et al., 2007; Kieffer et al., 2008; Samson et al., 2008; **Figure 1B**). It was also demonstrated that the α-helical region of CdvA supports membrane interaction, in synergy with ESCRT-III, which is responsible for the deformation of liposomes *in vitro* (Samson et al., 2011; **Figure 1**). These functions are comparable with the eukaryotic ESCRT system, where ESCRT-I and ESCRT-II facilitate scission of membrane at physiological concentrations of ESCRT-III (Wollert and Hurley, 2010).

*Sulfolobus acidocaldarius* produces three additional ESCRT-III-like paralogs (Saci_0451, Saci_1416, and Saci_1601) to form 40 nm wide membrane tubules, analogously to the eukaryotic system, after recruitment of ESCRT-III to the cell division site by CdvA (Samson and Bell, 2009; Samson et al., 2011). CdvA positions itself at the mid-cell region between segregated nucleoids, and its ring structures seem to constitute a platform for ESCRT-III rings between the nucleoids, supposedly to support their segregation. Furthermore, CdvA organizes into double-helical structures stabilized by DNA without any conventional DNA-binding motifs (Moriscot et al., 2011). The central region (residues 69–196) of CdvA in *Sulfolobus acidocaldarius* shows the signature of a coiled-coil motif for DNA binding and the formation of nucleoprotein filaments. The protein sequence of CdvA further suggests that the N-terminal 70 residues might form a β-barrel with similarity to the PRC-barrel, a widespread domain implied in a number of cellular processes (Anantharaman and Aravind, 2002).

Because the double helical structure of CdvA filaments resembles actin filaments, CdvA could be a functional analog of archaeal actin. Thus, both, the morphology of CdvA filaments, and the phylogenetic analyses, support the hypothesis that it constitutes an ancient cytoskeleton protein involved in cell division. But also the alternative proposition, that FtsA is an ancient cell division protein of the actin family, which evolved into a cytoskeletal element, should be mentioned (Sanchez et al., 1994). Moreover, the formation of such helical filaments fit well with observations of CdvA structures in the cell prior to nucleoid segregation (Samson and Bell, 2009; Samson et al., 2011).

## ESCRT-III, A MEMBRANE SCISSION COMPLEX

All clades of eukaryotes have ESCRTs (Dacks and Field, 2007). All Archaea having a Vps4-like ATPase and expressing ESCRT-III-like homologs form a closely related group, although they are lacking genes for ESCRT-0, ESCRT-I, or ESCRT-II. Moreover, one gene of an ESCRT-III-like subunit is always adjacent to the Vps4-like ATPase gene. This conserved gene cluster arrangement suggests that they encode for functional partners (Obita et al., 2007).

One major finding was that eukaryotic ESCRT-III proteins alone have the specific ability to drive the detachment of intraluminal vesicles (ILVs; Wollert et al., 2009a). For this purpose, ESCRT subunits are recruited from the cytosol to build up multivesicular bodies (MVBs), to facilitate budding of certain enveloped viruses, or to catalyze posterior steps of cytokinesis (Saksena et al., 2007; Hurley et al., 2009; Raiborg and Stenmark, 2009). ESCRT-III was identified as the minimal membrane fission machinery, which assembles a membrane neck structure during budding, and localizes between two separating daughter cells (Wollert et al., 2009a; Wollert and Hurley, 2010). Purified ESCRT-III subunits of *Saccharomyces cerevisiae* have been shown to facilitate budding and scission of ILVs from giant unilamellar vesicles (GUVs) at a high concentration level (Im et al., 2009; Wollert et al., 2009a).

Thus, the ESCRT-III complex obviously plays an important role in the constriction of the membrane neck structure required for complete fission (Peel et al., 2011). This ESCRT-III mediated membrane contraction resembles the membrane constriction by dynamin, but with different membrane topology. In the case of dynamin, helical structures assemble on the outside of the membrane neck to dissever endocytotic vesicles from the plasma membrane, leading to fission (Mettlen et al., 2009). In contrast, ESCRT-III seems to mount on the inner membrane neck and catalyze membrane rupture by the interplay with Vps4 ATP hydrolysis (Lata et al., 2008; Fabrikant et al., 2009; Wollert et al., 2009a; Wollert and Hurley, 2010; Peel et al., 2011). In *Sulfolobus solfataricus* it was shown that the C-terminal region and the residues 164–207 of ESCRT-III are essential for the direct interaction to the archaeal Vps4-like ATPase MIT domain (Obita et al., 2007).

## Vps4, A CELL DIVISION INVOLVED ATPase

The eukaryotic Vps4 is the first example of an AAA-type ATPase where the oligomeric structures are important for ATP hydrolysis (Babst et al., 1998). In *Saccharomyces cerevisiae*, endogenous Vps4 forms stable dimers, but in the presence of ATP, these dimers oligomerize into a stable, ring-like decameric complex of five symmetric dimers (Babst et al., 1998; Obita et al., 2007). Interestingly, two features of Vps4 are responsible for its cycling between dimeric and oligomeric forms: the nucleotide-dependent self-affinity of the Vps4 dimers, and the regulation of ATP hydrolysis by the oligomeric state.

Nucleotide binding and hydrolysis by the AAA domain directly modifies the structure of the ATPase Vps4, and also shifts the subcellular distribution of Vps4, ESCRT-III, and Vps32/Snf7 (Babst et al., 1998). It also seems that Vps4 binds to an endosomal compartment through a coiled-coil domain, interacting with a membrane-bound protein complex of Vps24 (ESCRT-III-like protein) or Snf7, both also containing coiled-coil domains (Takei et al., 1995). For this purpose, Vps4 dimers in the ATP-bound form are recruited from the cytosol to the endosomal membrane, which leads in turn to oligomerization of Vps4 to decamers, binding to the ESCRT-III complex (class E Vps protein complex) via coiled-coil interactions. The oligomerization of Vps4 dimers accessorily activates ATP hydrolysis, which results in a conformational change in the coiled-coil domain and in return in disassembly of the ESCRT-III complex (Babst et al., 1998).

In this context, eukaryotic Vps4 operates as an endosomal-bound dissociation factor for the ESCRT-III complex by its ATPase activity (Babst et al., 1998; Peel et al., 2011). Deletion mutants and siRNA studies revealed that all human ESCRT-III and Vps4 proteins are essential for the completion of cytokinesis (Carlton and Martin-Serrano, 2007; Morita et al., 2007, 2010; Dukes et al., 2008). In *Sulfolobus solfataricus*, the interaction between the MIT domain of the Vps4-like protein and the C-terminal end of the ESCRT-III-like protein was demonstrated (Obita et al., 2007; Hobel et al., 2008). The isolated Vps4 MIT domain reveals only a modest affinity to ESCRT-III, but in contrast, the dodecameric Vps4 complex binds its target efficiently (Obita et al., 2007). Both Vps4-interacting ESCRT-III-related subunits, Vps2 and Did2, are required for efficient vacuolar sorting (Nickerson et al., 2006).

On the ATP hydrolysis-induced transition of Vps4 to a monomer, the low affinity of a single MIT domain for ESCRT-III would facilitate the rapid dissociation of Vps4 from ESCRTs. The ESCRT-III complex triggers its own disassembly. It recruits monomeric or dimeric Vps4 to membranes, on which the multimeric ESCRT-III aggregate facilitates the assembly of the active Vps4 oligomer. Once the Vps4 rings have assembled, ATP hydrolysis would lead to the disassembly of both the ATPase rings and the ESCRT-III complex (Obita et al., 2007).

## RECONSTITUTION PERSPECTIVES

With respect to a possible reconstitution of the division process from the bottom-up, bacteria and Archaea promise more restricted and practically manageable modules, compared to the vastly complex cytokinetic machineries in eukaryotes. In recent years, our group has explored the reconstitution of the *Escherichia coli* cell division system, module by module. We successfully reproduced in vitro the emergence of self-organized waves, and lately, oscillations, of the Min protein machinery positioning the contractile Z ring (Loose et al., 2008; Zieske and Schwille, 2013). The MinCDE system does likely not take part in the constriction, but is an essential septum positioning device. *E. coli* min deletion mutants are still viable, but with fluctuating localization of cytokinesis (Teather et al., 1974; de Boer et al. 1989, 1990, 1992). We furthermore investigated the assembly of FtsZ proto-rings on membranes and their curvature sensing and inducing propensity (Arumugam et al., 2012), however with no indication so far that the contractile force required for cell division could result from FtsZ only. Several other research groups presently attempt the reconstitution and assembly of bacterial divisome, based mainly on FtsZ and its anchors, in vesicles (Osawa et al., 2008; Jimenez et al., 2011; Cabre et al., 2013; Osawa and Erickson, 2013). However, considering that a progressively contractile role of FtsZ could so far not be established, in spite of its membrane-sculpting ability, it seems appropriate to explore alternative membrane-transforming machineries to accomplish compartment division. Thus, we presently investigate in a cell-free system the interaction of the three archaeal cell division proteins discussed above: Vps4, ESCRT-III, and CdvA of *Sulfolobus acidocaldarius*. As a first step, we accomplished their intra- and extra luminal reconstitution into artificial constructed liposomes, in a bottom-up approach.

**FIGURE 2:**
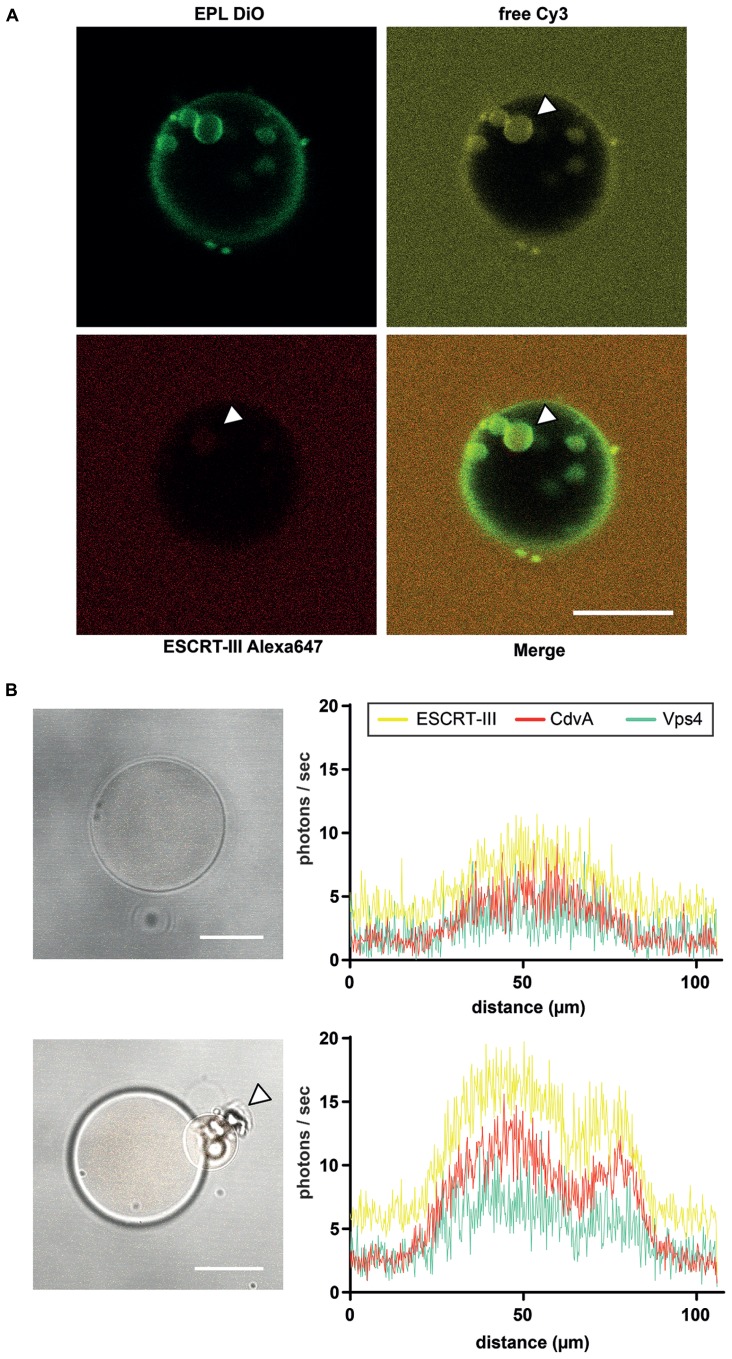
**Liposome preparation for intraluminal experiments. (A)** ILV formation into EPL (*Escherichia coli* polar lipids) GUVs in presence of ESCRT-III and Vps4. The GUV membrane is stained green with DiO. Red labeled ESCRT-III proteins (Alexa647), unstained Vps4 proteins and free Cy3 were added to the extraluminal buffer of the EPL GUVs. After ILV formation of the GUV membrane ESCRT-III as well as the soluble marker Cy3 could be visualized in the vesicles (white arrowheads). Scale bar, 10 μm. **(B)** *De novo* protein production in GUVs via protein synthesis kit within 3 h. Left figures reveal brightfield images of a GUV with recombinant CdvA-mCherry, Vps4-AmCyan1 and ESCRT-III-zYellow1 plasmids in combination with a protein synthesis kit included. The low fluorescence level (histogram in upper right) of all three proteins indicates the beginning of *de novo* production. After 3 h of incubation the fluorescence of all proteins are increased (histogram in lower right) and it could be observed a budding event at GUV (lower left image, white arrowhead). Scale bar, 10 μm.

Confocal imaging reveals extraluminal membrane binding and ILV formation into EPL (*Escherchia coli* polar lipids) GUVs in presence of ESCRT-III and Vps4 (**Figure 2A**). Intraluminal experiments with the ESCRT-III machinery were designed following the so-called droplet method, in which water-in-oil droplets are converted into liposomes through sonication and two-step centrifugation (Pontani et al., 2009; van Swaay and deMello, 2013). This method is compatible with the use of purified or *de novo* synthesized proteins within the liposomes. Thus, we may study the function and interplay of Vps4, ESCRT-III, and CdvA, either using purified proteins, or by intraluminal *de novo* protein synthesis. In both cases, the protein abundance can be visualized by confocal imaging via LSM780 microscope (Zeiss). Therefore, the genes of Vps4, ESCRT-III, and CdvA (saci_1372 to saci_1374) were ligated into recombinant fluorescence plasmids. Purified cell division proteins were labeled either C-terminally or N-terminally with one fluorophor (mCherry, Yellow1, or AmCyan1; **Figure 2B**). GUVs are useful model systems to reveal membrane transformation by the expressed proteins. Their mean diameter (1–50 μm) can be made comparable to the average size of *Sulfolobus acidocaldarius* cells (1–2 μm). Alternatively, 80–800 nm sized large unilamellar vesicles (LUVs) may be explored in prospective studies, which are, however, much less amenable to standard light microscopy observation.

Remarkably, specific deformation of GUVs after co-reconstitution of all three proteins could be observed reproducibly. This phenomenon will now be subject to a more quantitative analysis, addressing the range of shapes and structures formed on and from the membranes. We will address the influence of electric charge polarity and lipid composition of the membrane vesicles on curvature and general abundance of budding events. Further, the interactions of crenarchaeal ESCRT-III-like paralogs (Saci_0451, Saci_1416, and Saci_1601) should be investigated, to address the question whether or not these paralogs are essential, or only required for fine-tuning of cell division in *Sulfolobus acidocaldarius*. To access spatial scales below the optical resolution limit, we have begun to visualize the interaction of DNA with the CdvA complex, leading to its double helical structure, by super-resolution microscopy [stochastic optical reconstruction microscopy (STORM)] and atomic force microscopy (AFM).

Clearly, these data constitute only the beginning of a series of experiments, exploring the potential of ESCRT-III mediated (proto-) cell division in a minimal system. Besides the fundamental mechanistic aspects of membrane transformation that may be addressed by such a bottom-up reconstitution, also the phylogenetic and evolutionary history of the division machinery in Crenarchaeota may be explored. This may answer the questions on diversity of the FtsZ-based systems, and why ESCRT-III mediated cell division is now mainly found in thermophilic and acidothermophilic Crenarchaeota and Thaumarchaeota. The linkage between cell division in Archaea and ESCRT-III dependent biogenesis of MVBs in eukaryotes is a fascinating evolutionary aspect (Poole, 2006; Hanson et al., 2008). Taken together, we will explore the scaling variability of ESCRT-III mediated constriction, and potentially, facilitate ESCRT-III based division of larger membrane compartments, to reveal its potential suitability as a truly “archetypical” cell division machinery to be assembled from the bottom-up.

## Conflict of Interest Statement

The authors declare that the research was conducted in the absence of any commercial or financial relationships that could be construed as a potential conflict of interest.
